# Traumatic Brain Injury: A Perspective on the Silent Epidemic

**DOI:** 10.7759/cureus.15318

**Published:** 2021-05-29

**Authors:** Ali Alkhaibary, Abdulaziz Alshalawi, Raad M. M Althaqafi, Abdullah A Alghuraybi, Ali Basalamah, Ahmed M Shammaa, Ali A Altalhy, Tamer M Abdelrahman

**Affiliations:** 1 Division of Neurosurgery, Department of Surgery, King Abdulaziz Medical City, Ministry of National Guard - Health Affairs, Riyadh, SAU; 2 Neurosurgery, King Abdullah International Medical Research Center, Riyadh, SAU; 3 College of Medicine, King Saud bin Abdulaziz University for Health Sciences, Riyadh, SAU; 4 Department of Surgery, College of Medicine, Taif University, Taif, SAU; 5 Department of Neurosurgery, King Saud University Medical City/King Khalid University Hospital, Riyadh, SAU; 6 Department of Surgery, College of Medicine, Medical University of Warsaw, Warsaw, POL; 7 Department of Neurosurgery, King Faisal Medical Complex, Taif, SAU

**Keywords:** collision, coma, head, injury, traffic

## Abstract

Background: Epidemiological data regarding the causes, patterns, severity, and outcomes of traumatic brain injury (TBI) are essential to plan for preventive strategies addressing this public health epidemic. The main aim of this study is to explore the patterns and causes of traumatic brain injury at two trauma centers.

Methods: A retrospective cohort study was conducted using a pre-tested validated data collection sheet. Data were collected from the medical records and electronic database of patients who presented to the emergency department with head trauma. Variables including the mechanisms, patterns of the injury, accompanying injuries, level of consciousness, and hospitalization duration were investigated for any possible association.

Results: A total of 269 patients (78% males, 22% females) who satisfied our study criteria were included in the final analysis. Motor vehicle collisions were the most common reason for traumatic brain injury (57.6%) followed by falls (28.3%). There was a statistically significant association observed between type of hemorrhage and Glasgow coma scale at initial presentation (P < 0.05).

Conclusion: The most common cause of traumatic brain injury is motor vehicle collisions, followed by falls. The public should be made aware of the importance of using safety and precautionary measures to minimize the impact of traumatic brain injuries. Educational programs for neurotrauma prevention can be developed and utilized as a blueprint for local hospitals and officials in the country.

## Introduction

Traumatic brain injury (TBI) is a silent public health epidemic and a major cause of disability, morbidity, and mortality worldwide [1]. TBI is characterized by a breakdown in the normal function of the brain caused by collision, blow, and jolt to the head-neck-spinal cord, possibly leading to temporary or permanent impairment [2]. Global epidemiological data show that approximately 69 million people suffer from TBI yearly. The highest incidence is reported in high-income countries compared to low-middle income countries [3].

Road traffic accidents are the leading cause of TBI. Higher rates of TBI in the United States are observed among older adults (2,232 per 100,000), followed by young children (1,591 per 100,000) [4]. Although there is scarcity in the data regarding TBI from the Kingdom of Saudi Arabia, the reported incidence is 116 per 100,000 [5]. In Saudi Arabia, approximately 74% of the cases of hemiplegia, paraplegia, and quadriplegia are due to motor vehicle collision (MVC) [6].

Due to the paucity of studies concerning TBI, the present study aimed to review the demographic information, mechanism of injury, severity of TBI, radiological findings on brain CT scan, length of stay (LOS), and the clinical/practical factors in traumatic brain injuries. 

This manuscript was presented orally at the Annual Meeting of the Saudi Association of Neurological Surgery on 1/3/2020.

## Materials and methods

Study setting

A retrospective cohort study was conducted using data collected from the medical records and electronic database of patients who presented to the emergency department with head trauma from April 2016 to April 2019. The study was conducted at two medical institutions, King Faisal Medical Complex and King Abdulaziz Specialized Hospital. These institutions provide medical care and advanced trauma care services to patients mainly from the Western region of Saudi Arabia. Data were collected and entered into a pre-designed and validated data collection sheet.

Patient eligibility

All patients who presented to the emergency department with traumatic head injuries during the study period were included. Initially, the records of 418 patients admitted to these two hospitals due to TBI were assessed. Patients with incomplete records and unsalvageable data were excluded from the analysis (35%). These included missing charts, unrecognizable radiological records, and incomplete documentation of post-traumatic symptomatology.

Data collection

The examined variables included socio-demographic details, mechanism of injury, accompanying injuries, Glasgow coma scale (GCS) on admission, radiological findings on brain CT, length of stay (LOS), and symptomatology. The severity of TBI was assessed using the GCS. TBI was classified into mild (GCS = 15-13), moderate (GCS = 12-9), and severe (GCS = 8-3). The association between the mechanism of injury and gender was analyzed.

Statistical analysis

Data were entered into Microsoft Excel by an investigator. Statistical analysis was performed using SPSS version 23 (IBM Corp., Armonk, NY, USA) by an independent biostatistician. Categorical variables were summarized as proportions and frequencies. Any possible association of the variables was analyzed using Pearson’s Chi-square test. Continuous variables were expressed as mean and standard deviation. A P-value of ≤ 0.05 was considered statistically significant.

Ethical considerations

The ethical committees of the Research and Studies Department, Directorate of Health Affairs approved the current study. Prior to commencement of the study, permission was obtained from King Faisal Medical Complex and King Abdulaziz Specialized Hospital. Patient-related data were preserved and kept confidential. The identifying personal details were not included in the analysis. The assigned protocol number is HAP-02-T-067.

## Results

Baseline characteristics

**Figure 1:**
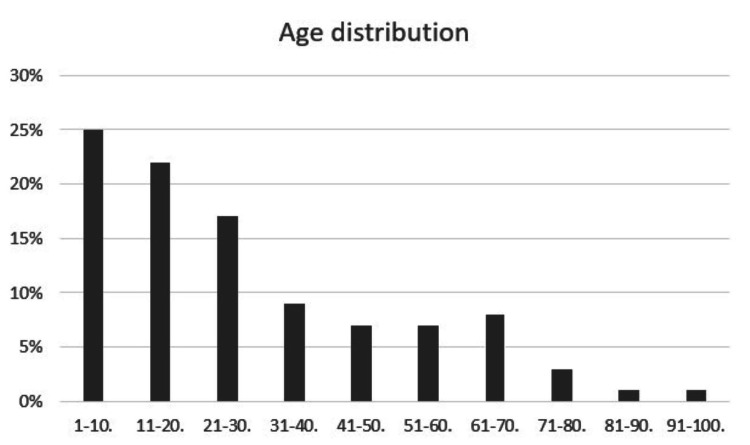
Age distribution of patients with traumatic brain injury presenting to the emergency department.

After scrutinizing the data, a total of 269 patients were included in the final analysis. The analysis included 22% females and 78% males who had some degree of TBI. The age distribution of the patients is illustrated in Figure 1.

Co-existing injury vs. type of hemorrhage 

When the co-existing injuries were assessed, it was found that 17.9% (n=10) of the patients with intra-axial hemorrhage had head and face injuries. Spinal cord injuries were noted in 5.7% (n=5) of the patients with extra-axial hemorrhage. Table 1 outlines the type of hemorrhage and co-existing injuries.

**Table 1:** Characteristics of the type of hemorrhage and the co-existing injuries. ND: Not defined.

Hemorrhage			Co-existing Injury									Total
			Abdomen & pelvis	Spinal cord	Chest	Extremities		Head & face	Mixed	ND	None	
	Intra-axial	N	0	0	2	2	10		3	5	34	56
		%	0.0%	0.0%	3.6%	3.6%	17.9%		5.4%	8.9%	60.7%	100.0%
	Extra-axial	N	1	5	0	3	10		8	12	48	87
		%	1.1%	5.7%	0.0%	3.4%	11.5%		9.2%	13.8%	55.2%	100.0%
	Intra and extra-axial	N	0	1	1	0	2		3	7	20	34
		%	0.0%	2.9%	2.9%	0.0%	5.9%		8.8%	20.6%	58.8%	100.0%
	None	N	1	2	3	3	9		6	8	60	92
		%	1.1%	2.2%	3.3%	3.3%	9.8%		6.5%	8.7%	65.2%	100.0%
Total		N	2	8	6	8	31		20	32	162	269
		%	0.7%	3.0%	2.2%	3.0%	11.5%		7.4%	11.9%	60.2%	100.0%

Mechanism of injury vs. gender

TBI was more common in males than females. Motor vehicle accidents in males accounted for 45.72% (n = 123) of trauma patients presenting to the emergency department. Table 2 outlines the mechanism of injury according to the gender of the patient.

**Table 2:** Patterns of the mechanism of injury according to the gender of the patient. MVC: Motor vehicle collision.

		Mechanism of Injury							
Variable		Pedestrian	MVC	Motorcycle	Bike	Falls	Struck by objects	Assault	Total
Gender									
	Male	5	123	5	3	57	1	16	210
	Female	1	32	4	0	19	2	1	59
Total		6	155	9	3	76	3	17	269

Type of hemorrhage vs. Glasgow coma scale

According to the GCS at initial presentation to the emergency department, 15.2% of the patients had severe TBI, 14.1% had moderate TBI, and 70.6% had mild TBI. When the type of hemorrhage was assessed, it was found that 19.5% of the patients with intra-axial hemorrhage had mild TBI. On the other hand, 28.9% of the patients with moderated TBI had extra-axial hemorrhage. The association between the type of hemorrhage and GCS was statistically significant (P = .001). Table 3 outlines the association between the type of hemorrhage and the severity of GCS.

**Table 3:** Association between the type of hemorrhage and Glasgow coma scale.

Glasgow coma scale			Hemorrhage				Total	P-value
			Intra-axial	Extra-axial	Intra and extra-axial	None		
	Mild (15-13)	N	37	64	15	74	190	0.001
		%	19.5%	33.7%	7.9%	38.9%	100.0%	
	Moderate (12-9)	N	10	11	6	11	38	
		%	26.3%	28.9%	15.8%	28.9%	100.0%	
	Severe (≤ 8)	N	9	12	13	7	41	
		%	22.0%	29.3%	31.7%	17.1%	100.0%	
Total		N	56	87	34	92	269	
		%	20.8%	32.3%	12.6%	34.2%	100.0%	

Mechanism of injury vs. fracture type

It was observed that a fracture of the vertebrae was more prevalent in MVCs. Fractures involving the base of the skull were noted in those who had a fall. Fractures involving the vault of the skull were more prevalent in assault. Table 4 outlines the type of fracture and the reason for injury.

**Table 4:** Characteristics of the type of fracture and the mechanism of injury. MVC: Motor vehicle collision. ND: Not defined.

		Mechanism of injury						
Variable		Pedestrian	MVC	Motorcycle	Bike	Falls	Struck by objects	Assault
Fracture								
	Facial	0	6	2	0	2	2	2
	Skull vault	0	20	1	0	9	0	6
	Skull base	1	2	1	0	5	0	0
	Vertebra	1	6	0	0	0	0	0
	Mixed	0	6	0	0	7	0	0
	None	3	96	5	3	46	1	6
	ND	1	19	0	0	7	0	3
	Total	6	155	9	3	76	3	17

Type of fracture vs. hemorrhage 

Fracture of the vault of the skull was noted in 17.2% (n=15) of the patients with extra-axial hemorrhage. It was observed that a fracture of the vertebrae was more prevalent in patients with simultaneous intra- and extra-axial hemorrhage. Table 5 outlines the patterns of the type of hemorrhage and the type of fracture.

**Table 5:** Patterns of the type of hemorrhage and type of fracture.

Hemorrhage			Type of fracture							Total
			Vault of the skull	Base of the skull	Vertebra	Complex	Facial bones	ND	No fracture	
	Intra-axial	N	7	1	1	2	3	6	36	56
		%	12.5%	1.8%	1.8%	3.6%	5.4%	10.7%	64.3%	100.0%
	Extra-axial	N	15	6	2	7	4	14	39	87
		%	17.2%	6.9%	2.3%	8.0%	4.6%	16.1%	44.8%	100.0%
	Intra and extra-axial	N	7	1	3	2	2	3	16	34
		%	20.6%	2.9%	8.8%	5.9%	5.9%	8.8%	47.1%	100.0%
	None	N	7	1	1	2	5	7	69	92
		%	7.6%	1.1%	1.1%	2.2%	5.4%	7.6%	75.0%	100.0%
Total		N	36	9	7	13	14	30	160	269
		%	13.4%	3.3%	2.6%	4.8%	5.2%	11.2%	59.5%	100.0%

Length of stay vs. type of hemorrhage

The analysis of CT scan findings showed that 20.8% of the patients had intra-axial hemorrhage, 32.3%% (n=87) had extra-axial hemorrhage, and 12.6% had simultaneous intra- and extra-axial hemorrhage. When the association between the type of hemorrhage and the length of stay was assessed, it was found that most patients (n = 196; 66.2%) were hospitalized for less than one week (P = 0.007). Table 6 outlines the association between type of hemorrhage and the length of stay during hospitalization.

**Table 6:** Association between the type of hemorrhage and length of stay during hospitalization *Significance level <0.05 †The length of stay during hospitalization is measured in days.

Hemorrhage		Length of Stay†		Total	P-value*
		≤ 7	>7		
Intra-axial	N	37	19	56	.007
	%	66.1%	33.9%	100.0%	
Extra-axial	N	66	21	87	
	%	75.9%	24.1%	100.0%	
Intra and extra-axial	N	18	16	34	
	%	52.9%	47%	100.0%	
None	N	75	17	92	
	%	81.5%	18.4%	100.0%	

## Discussion

Reports regarding TBI vary widely between and within countries due to the lack of data regarding the types, causes, and outcomes of TBI. The present study is a hospital-based, retrospective cohort analysis conducted at two medical institutions in Saudi Arabia. The findings of the study demonstrated that motor vehicle collisions (MVCs) were the leading cause of TBI. This concurs with other studies conducted in Middle Eastern countries, i.e. the United Arab Emirates and Qatar [7-8].

In contrast, a study in the United States reported that falls from heights and assault were the leading causes of TBI. However, MVCs remained the leading cause of death, followed by falls from heights [9]. In the present study, most patients with TBI were males and the leading cause of TBI in males was MVCs, although there was no significant association. This could be due to the fact that females were not allowed to drive during the major period of our study compared to other countries [7-9].

Associated injuries 

Brain injuries resulting from MVCs, assault, or falls from heights frequently cause concurrent injuries to other body regions, including the spine, vertebrae, and extremities, the leading cause of death and disability [10]. The findings of the present study showed that head and spine injuries were commonly noted in MVCs and physical assaults. Studies show that one-third to one-half of the patients with severe extra-cranial injuries with associated brain injury have high mortality rates [11-13]. The reason for this could be explained on the basis that patients with injury at the extra-cranial sites could have massive hemorrhage, which could result in coagulopathy or decreased cerebral blood flow, causing secondary brain damage [14-15].

Severity of brain injury 

The GCS is commonly recorded soon after arrival or within less than one hour after arrival to the hospital [16]. In our study, 15.2% of patients had severe brain injury whereas 14.1% had moderate brain injury. It was found that the severe brain injuries were noted in patients who had fractures of the vault of the skull, base of the skull, and vertebrae compared to other types of fracture. This finding was in accordance with the findings of Meng and Shi [17].

In another study conducted in the United States, it was reported that 14.5% of the patients who had severe brain injury had achieved good outcome at six months [18]. Studies also reported that 100% of mortality was observed in cases with fixed and dilated pupils who had severe brain injury [19-20].

Hospitalization 

It is reported that patients with intracerebral hemorrhage (ICH) due to trauma suffer from long and uncertain hospitalization [21]. Therefore, it is important to take this into consideration to predict the outcomes and help healthcare providers take precautionary measures when managing patients with TBI. Studies report that various injury preventive programs were effective in reducing mortality and disability in patients who had road traffic accidents [22-23].

Age at risk

In the present study, the majority of victims with TBI (47%) were 20 years of age and younger. This emphasizes the issue within the affected demographic. Similarly, El-Matbouly et al. conducted a retrospective study on TBI across age groups in Qatar. Their findings showed that the majority of victims were young adults (34%) and middle-aged (21%). This concurs with the findings of the present study. Therefore, young adolescents should be properly made aware of traumatic brain injury and how it can be a silent epidemic [24].

Concurrent maxillofacial injury 

Aldwsari et al. prospectively explored maxillofacial traumas in patients who sustained road traffic accidents from 2013 to 2018. The authors highlighted the important association of maxillofacial traumas with traumatic brain injuries. Of note, the face is the most susceptible area to trauma by road traffic accidents, being the most exposed area of the body. In the study of Aldawsari et al. approximately 60% of the cohort who sustained maxillofacial trauma suffered from concomitant brain damage [25].

Initial assessment 

TBI could be associated with loss of cognitive, physical, or psychosocial functions leading to decreased level of consciousness, memory impairment, neurologic deficits, and alteration in the mental state of the individual [26]. Most of the MVCs are considered emergency cases and the first and foremost protocol in management is the maintenance of airway, breathing, circulation, stabilization of the disability, and prevention of secondary brain injury. In this situation, the initial assessment includes resuscitation and neurological examination [27].

Prognosis 

The management of severe TBI is complex and overwhelming. Although several prognostic models have been developed and tested, the accurate assessment of short and long-term prognosis remains poorly validated. There is a need to properly understand the molecular mechanisms of TBI, which would help healthcare providers to accurately predict the outcomes [28].

Limitations 

Our study was retrospective and hospital-based, hence could have some limitations of its own. There is a chance of selection bias and misclassification that could arise while retrospectively processing the data. Second, the type of management the patient received during hospitalization was not detailed, which might influence recovery. Despite these limitations, the present study highlighted that motor vehicle accidents and falls were the leading causes of TBI, thus emphasizing the importance of considering the implementation of preventive measures and appropriate public health programs including traffic safety rules. This helps to develop educational programs for neurotrauma prevention to be utilized as a blueprint for local hospitals and officials in Saudi Arabia.

## Conclusions

The leading cause of traumatic brain injury in Saudi Arabia is motor vehicle accidents, followed by falls. The public should be made aware of road traffic rules and safety practices in motor vehicle accidents. People working at heights, such as building construction workers, have a high potential for falls and should be properly instructed to take precautionary and safety measures, i.e., protective equipment, in order to reduce the impact of traumatic brain injury.
